# Supplementary material for: Dynalign II: common secondary structure prediction for RNA homologs with domain insertions
Source: Nucleic Acids Res. 2014 Nov 21;42(22):13939–48. doi: 10.1093/nar/gku1172 (PMC4267632; doi:10.1093/nar/gku1172)
Supplement: SUPPLEMENTARY DATA [file supp_gku1172_nar-02021-z-2014-File012.zip › manual/Text/Building.html]

RNAstructure Command Line Help: Building the Interfaces


|  |  |  |
| --- | --- | --- |
|  | RNAstructure Command Line Help Building Instructions | - Contents - Index |
| Do I need to build the package?  RNAstructure is provided both as source code and as prebuilt executables. Prebuilt executables are currently available for 32-bit Linux, 64-bit Linux, Max OS-X (Leopard and higher), and 32-bit Windows. If you are using one of these operating systems, then you probably do not need to build the package. The available executables will work fine.   Configuring the Build on Unix/Linux/OS-X  The command line (text) interfaces for RNAstructure are built using the Makefile in the main directory (RNAstructure/). The definitions for compiler and flags are in RNAstructure/compiler.h. This file is provided with the defaults set for compiling on Linux using g++. To change the platform and/or compilers, this file should be manually edited. Example flags are provided for Linux, Mac, and Cygwin platforms. To change the operating system, simply remove the "#" symbol (which denotes comments) from the variable definitions under the target type. Also, add "#" in front of the Linux flags.   Building on Unix/Linux/OS-X  To build the package, use the command "make all" in RNAstructure/. The command line executables are built and placed in RNAstructure/exe/. You should probably then update your PATH variable to include this directory.  An optional "make install" command will copy all the executables to the /usr/local/RNAstructure/ directory. This is helpful in a shared computing environment. This command can only be executed as root.   Building on DOS/Windows  For DOS/Windows command line interfaces, Microsoft Visual Studio (2005 or later) AND the Intel C++ compiler are reqired. Use Visual Studio to open the solution called RNAstructure\RNAstructure\_windows\_text\_interfaces\RNAstructure\_windows\_text\_interfaces.sln. Then build using Visual Studio. The executables will be depositing in the RNAstructure\RNAstructure\_windows\_text\_interfaces\Release folder. | | |
| Visit The Mathews Lab RNAstructure Page for updates and latest information. | | |
